# Towards a Measure for Characterizing the Informational Content of Audio Signals and the Relation between Complexity and Auditory Encoding

**DOI:** 10.3390/e23121613

**Published:** 2021-11-30

**Authors:** Daniel Guerrero, Pedro Rivera, Gerardo Febres, Carlos Gershenson

**Affiliations:** 1 Posgrado en Ciencia e Ingeniería de la Computación, Universidad Nacional Autónoma de México, Mexico City 04510, Mexico; 2 Centro de Ciencias de la Complejidad, Universidad Nacional Autónoma de México, Mexico City 04510, Mexico; pedro.rivera@c3.unam.mx (P.R.); cgg@unam.mx (C.G.); 3 Departamento de Procesos y Sistemas, Universidad Simón Bolívar, Sartenejas, Baruta, Miranda 1080, Venezuela; gerardofebres@usb.ve; 4 Instituto de Investigaciones en Matemáticas Aplicadas y Sistemas, Universidad Nacional Autónoma de México, Mexico City 04510, Mexico; 5 Lakeside Labs GmbH, Lakeside Park B04, 9020 Klagenfurt am Wörthersee, Austria

**Keywords:** multiscale complexity, entropy, information content, auditory encoding, music

## Abstract

The accurate description of a complex process should take into account not only the interacting elements involved but also the scale of the description. Therefore, there can not be a single measure for describing the associated complexity of a process nor a single metric applicable in all scenarios. This article introduces a framework based on multiscale entropy to characterize the complexity associated with the most identifiable characteristic of songs: the melody. We are particularly interested in measuring the complexity of popular songs and identifying levels of complexity that statistically explain the listeners’ preferences. We analyze the relationship between complexity and popularity using a database of popular songs and their relative position in a preferences ranking. There is a tendency toward a positive association between complexity and acceptance (success) of a song that is, however, not significant after adjusting for multiple testing.

## 1. Introduction

Despite sound’s intrinsic complexity, the human brain can decode and process it to extract valuable information from its environment. The brain can estimate distances, roughly identify the materials producing a specific sound, and even estimate the number of objects producing the sound [1,2]. The brain also assesses the different sounds it perceives and orders them according to our preferences. When hearing a sound, it is easy to classify it as pleasant or unpleasant. Even when the precise elements and processes involved in this decision are not clear, we are perfectly conscious of the final result of this evaluation.

In particular, when our brain listens to music, it performs a classification process, and this classification is made based on the intrinsic properties associated with music. We can say that these intrinsic properties conform to music’s informational content. Some authors [3] support the idea that sound preferences are dominated by a trade-off between the simple and the complicated (the expected and the unexpected elements, regular or random). When a song is too simple, it does not generate the necessary stimuli to maintain the listener’s attention. On the other hand, if the song is too complicated, in the sense that it does not offer recognizable patterns and too dense information is required to describe it, just as noise is, it is not attractive either. This suggests the existence of an intermediate, “optimal” balance between these two extremes. There have been several proposals to measure and characterize the informational content of a musical segment. These approaches range from analyzing the motifs of the network associated with the transition between notes in a song [4], to the analysis of the underlying language in digital format [5]. Nevertheless, there is no clear definition to capture the complexity and informational content of a song.

The present article explores the relationship between complexity and preferences using music as our object of study. To achieve this, we define a metric to characterize the complexity of a musical segment. Then we explore to what extent the complexity of a song affects the degree of acceptance. We evaluate the multiscale entropy as a candidate to characterize the complexity associated with the melody of a musical segment. Specifically, we study the correlation between multiscale entropy and the listener’s preferences considering pitch intervals (in the musical sense) at different periods of a song.

The paper is structured as follows. In Section 2, we survey the most relevant literature on the relationship between music and complexity and describe the different approximations to the problem. Section 3 describes the complexity metrics we use, the data, and also the processing transformations involved. Section 4 presents the most important findings derived from our analysis. Finally, Section 5 provides a summary of the contributions and limitations of our work. We end with some proposals for future work.

## 2. Background and Related Work

In 2015, Febres et al. [6] computed the informational entropy of languages applying Shanon’s proposed information metrics: entropy [7]. This assessment of language’s information used words as the symbols making up languages. Later, Febres and Jaffe [5] applied similar ideas to determine the information content of the songs. Since there were no words, in this case, the authors analyzed the information content of music by using the language associated with the Music Instrument Digital Interface (MIDI) format. This language contains all the necessary instructions to generate and reproduce the specified song. With this language, it was possible to estimate the informational entropy, among other useful metrics, and characterize the associated information content of the songs. This characterization makes it possible to identify the musical genre and analyze changes in music’s complexity over time.

In the work of Perez-Verdejo et al. [8], they analyze music consumption patterns in Mexico using streaming statistics and audio features from the music streaming platform Spotify. The authors investigate how music features correlate with the streaming metric and compare the regional (Mexican) patterns with global (worldwide) counterparts. The authors identify the features that clearly distinguish or characterize the most popular songs in Mexico.

In 2014, Gamaliel et al. [9] introduced the concept of instrumentational complexity and showed that there exists a relationship between instrumentational complexity and album sales. They found a negative association between complexity and sales. The conclusion is that the simpler albums (measured by their metric) tend to be associated with higher sales: simplicity sells. This measure of instrumentational complexity is based only on the number and the uniqueness of the instruments used in the song. From an information-theoretical point of view, this metric is not genuinely associated with the informational content of a musical segment. In our opinion, a measure for musical complexity must consider the intrinsic elements of the music.

In their work, Parmer et al. [10] analyze popular songs and classify them by their associated complexity. By transforming each song into a sequence of tokens, they generate a language. Then, the authors use a conditional version of Shannon’s entropy [7] to measure the complexity of a song expressed as a sequence of tokens. They found an inverted-U-shaped relationship between popularity and entropy. With this characterization, they identify the musical genre of the songs based on their entropy profile.

In Overath et al. [11] they show that brain activity in the *Planum Temporale* (a brain region typically associated with audio processing) when measured via functional Magnetic Resonance Imaging (fMRI) is positively associated with the complexity of the incoming auditory stimulus. The authors generate a series of pitch sequences with a pre-specified entropy and analyze the exhibited level of activity in the brain’s response. They show that when the entropy of the audio signal is high, so is the activity in the *Planum Temporale*, so there is a positive relation between signal complexity and brain activity.

In the present study, we follow the work of Carpentier et al. [12]. In this article, the authors explore the relationship between the complexity of the environment (input) and the complexity in the associated brain response (processing/decoding). A group of participants is exposed to a series of auditory stimuli while asked to perform a perceptual or emotional task. The activity in the brain response for each task is measured via fMRI. The aim is to evaluate whether the association between the stimulus’s complexity and the response’s complexity (complexity matching) explains the listener preferences. The authors found higher complexity matching during perceptual music listening tasks compared to emotional music listening tasks. This analysis is, to some extent, related to Ashby’s law of required variety [13,14] in the sense that, in order to process a complex signal, the brain must be able to use an at least equally complex decoding process. To characterize these complexities, both of the input and the brain activity, the authors use multiscale entropy.

## 3. Materials and Methods

It is generally understood that a complex phenomenon lives in an intermediate point between chaos and regularity [15]. However, none of these perfectly describes a complex process. Intuitively, complexity is associated with structural richness and the meaning of the underlying process.

**Figure 1:**
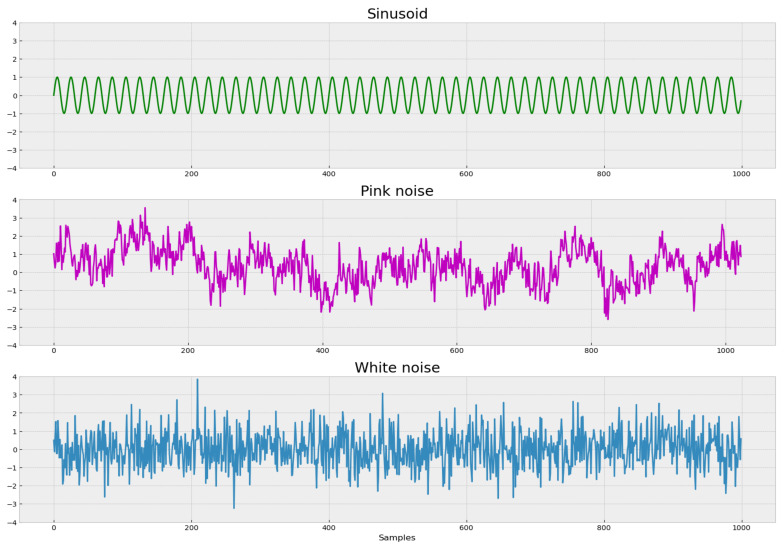
Three signals with different structural properties: sinusoidal, pink noise ( 1/f noise) and white noise.

As an example of how the complexity of the process is related to its regularity patterns, we analyze three signals: sinusoidal, pink noise ( 1/f noise) and white noise. Each of these processes has different structural properties and consequently different levels of complexity (Figure 1).

We use multiscale entropy (MSE) as our measure of complexity. Before applying MSE to music analysis, we investigate some of its properties on the three signals described above. MSE is a measure designed for the analysis of time series and one of its most important features is that it allows for evaluation across many different process scales. As described in the works of Siegenfeld et al. [16], Allen et al. [17], Bar-Yam [18] and Febres [19], the complexity depends on the scale at which the observer interprets the system. For a process to be complex, the interdependence between its elements must hold over the different scales of observation, not only at the extreme detailed system’s description. MSE allows this inter-scale analysis.

In addition to its mathematical properties, the other important motivation for selecting MSE as our complexity metric is that it has been applied to describe and characterize cognitive processes in experimental settings [20,21,22,23].

Based on the current literature and data availability, MSE is promising for exploring the relationship between complexity and preferences when applied to audio or musical analysis in particular. MSE is itself based on sample entropy (SE), which is a measure of the degree of compressibility of a signal [24,25]. The more compressible a signal is (fewer bits needed to represent it), the less its measure of SE. The intuitive definition of SE is clearly related to Siegenfeld et al.’s [16] definition of complexity and the notion of Kolmogorov Complexity [26]. SE is defined in the following terms for a series *S* consisting of *N* elements:

(1) $$SE=-log\left(\frac{S_{r}\left(m+1\right)}{S_{r}\left(m\right)}\right),$$

where $S_{r}\left(m+1\right)$ is the number of pairs of subsequences of size m+1 with distance less than *r*, and $S_{r}\left(m\right)$ is the number of subsequences of size *m* with distance less than *r*. The distance parameter *r* is set to 20% (following [24,27]) of the standard deviation of the full series *S*, and we use Euclidian distance.

SE algorithmically computes the conditional probability that, given a sequence of length *N*, any pair of subsequences with *m* similar consecutive points will also be similar in the m+1 point. SE is therefore a measure of self-similarity. The more self-similar the series is, the more redundancy it contains and the less its SE value. Note that, by construction, $S_{r}\left(m+1\right)$ will always be smaller than or equal to $S_{r}\left(m\right)$ (as adding a restriction can only reduce the number of coincidences), and therefore, SE will be greater or equal than zero (zero when the series is absolutely redundant).

However, SE does not fully capture the concept of complexity. It does not take into account the different scales involved in the process and it assigns high values (high complexity) to random processes. A white noise, while being not compressible, will obtain a high value of SE. For this reason, MSE should be introduced.

To calculate MSE from SE, it is necessary to apply a reduction process where the elements of the original series $S=\{s_{1},...,s_{n}\}$ are aggregated to create a unique element of the reduced series $Y^{\tau }=\left\{y_{1}^{\tau },...,y_{n}^{\tau }\right\}$ .

(2) $$y_{i}^{\tau }=\frac{1}{\tau }\sum_{i}^{i+\tau }s_{i}\;,$$

where τ∈{1,2,3,...} represents the scale of aggregation (number of aggregated elements).

SE ${}^{\tau }$ is calculated for each new series $Y^{\tau }$ while varying the parameter τ . These multiple SE ${}^{\tau }$ calculations, when taken altogether, represent the MSE metric.

If we now calculate MSE and SE for two known signals, white noise and pink noise, we observe that these measures attain different results for the same pair of signals.

Table 1 shows values of SE for a sinusoid, white noise, and pink noise. Even though pink noise has a richer structural complexity [15,28,29], white noise shows a higher SE value complexity.

**Figure 2:**
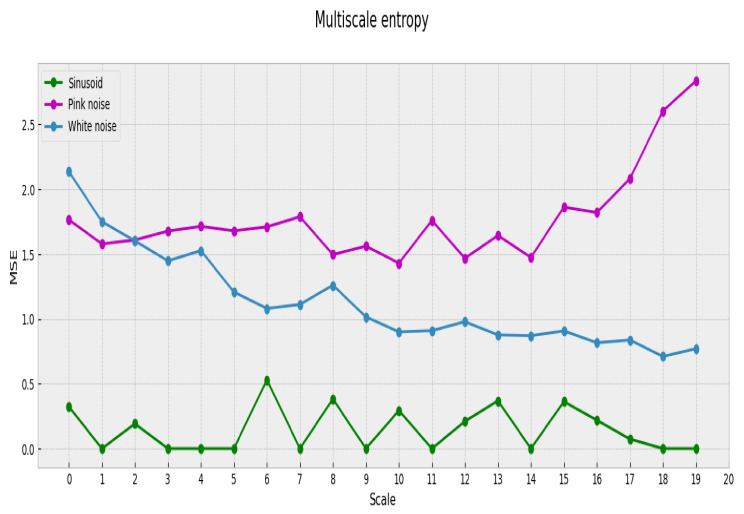
MSE for a sinusioidal signal, pink noise, and white noise.

If we calculate MSE for the signals mentioned above, we obtain not a scalar but a profile (the complexity profile) that represents the associated complexity. This profile spans through each of the considered scales (20 in our case), as shown in Figure 2.

Now it becomes clear that the estimated complexity for white noise is not consistent among all aggregation scales [17]. When the scale of aggregation augments, the white noise process reveals a simple structure that is not easily observed in the original scale. Instead, pink noise maintains an almost constant complexity among all scales and therefore is more complex than white noise. Based on its underlying properties, MSE offers a good approximation to the intrinsic complexity of a time series. We propose to use MSE in our analysis of the relation between complexity and preferences based on the following:

1. This metric is used to measure the complexity associated with brain processes. In particular, it is used to analyze the temporal activation patterns in specific brain regions. [20,21,22,23]
2. It allows the analysis of time series, such as music, over different observation scales.

Based on these considerations, MSE can provide useful insights in analyzing the relation between the informational content of a musical stimulus and the cognitive processes involved in the determination of musical preferences.

### 3.1. Data

#### 3.1.1. Music

The data sample used is part of the Million Song Dataset (MSD) [30], consisting of one million annotated songs (http://millionsongdataset.com/ (accessed on 30 September 2021)). Some of the included tags are *year of release*, *genre*, *album*, *artist* and a set of technical features per song. It is worth mentioning that the songs are already processed, there is no audio for the songs in the database, and only the extracted features are available. Table 2 shows some of the technical features included in the database.

Following the work of Overath et al. [11], we use the pitch component as the fundamental element of our analysis. It is also important to remark that the pitch is one of the perceptual components of music. Therefore, there is no strict relation between the physical properties of sound and our perception of pitch [2,31] (although it is related to the frequency component). The brain determines our perception of this component, which is why it is considered a relevant element for our analysis of musical preferences.

For each song, we use the component denominated *segment_pitches*. This component is a matrix of shape (*chroma_feature*, *time_segments*) that determines the relative presence of each pitch class in the corresponding time interval. This matrix is called the chromatogram of the song and represents the basic melody of the song.

#### 3.1.2. Music Preferences

To analyze the listener’s preferences we use the *year-end HOT 100 charts Billboards* https://www.billboard.com/charts/year-end/2020/hot-100-songs (accessed on 30 September 2021). These are a compendium of the most popular songs for each year in the United States and this ranking serves as a proxy for musical preferences. The basic idea is that these top songs have specific characteristics that make them different from the other songs and separate them into two sets: high popularity and low popularity.

### 3.2. Data Processing

MSE is meant to be used for time series, but our data are in matrix form. For that reason, we need to apply specific data processing steps to transform the matrix data into time series. The data processing steps are:

1. For each time segment, the most representative pitches are identified.
2. The original values of the matrix are mapped into the integer interval x∈[1,12]⊆Z .
3. Finally, the pitch dimension is collapsed to end up with a flattened matrix, i.e., a vector representing a time series of length time_segments .

**Figure 3:**
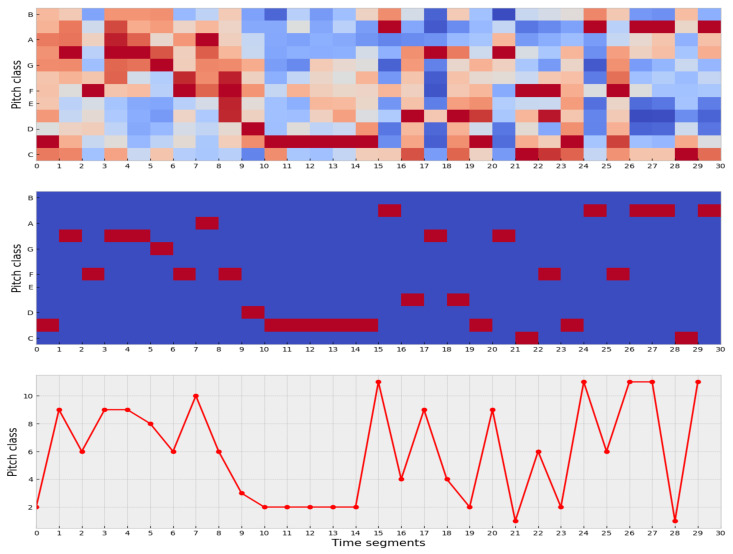
Transformation from a chromatogram matrix to a time series.

The intuition behind these transformations is that in each time segment, we seek to preserve only the most representative pitch for that time segment. In this way, the matrix representing the structure of the song reduces to its most representative perceptual element in each time segment. Figure 3 illustrates this process.

We obtain a time series for each song with these transformations, and now it is possible to calculate its corresponding MSE.

In order to create a proxy for the listener’s preferences, we use songs from the Billboards Hot 100 list. This list includes the 100 most-listened songs for each year in the United States and a ranking for the song’s popularity. Since the MSD database includes songs in the range of years from 1931 to 2011, we could analyze the differences in complexity between the songs included in the Hot 100 list and those not included for each year. Because MSE generates a complexity profile associated to each song, it is possible then to compare the complexity profiles of both groups and to determine if there is a significant difference between “successful” and “unsuccessful” songs.

## 4. Results

We use a sample of songs between the years 2000 and 2010. The 100 top songs are identified for each year, and its MSE is computed. MSE is also calculated for the songs not included in the Hot 100. Therefore, for each year, it is possible to separate the songs into two groups, successful and unsuccessful songs, and compare the complexity in each group.

**Figure 4:**
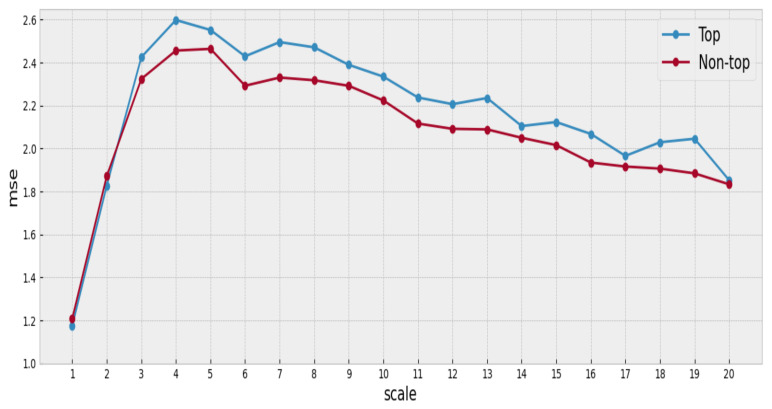
MSE for the year 2000.

The analysis of each time series includes scales from 1 to 20. The average is used to aggregate the complexity values per series over the appropriate scale. Figure 4 summarizes the findings of our analysis, and Appendix A includes the complete results and figures for other years.

We observe that the mean complexity is higher for songs belonging to the top group at most scales, suggesting that the songs with a better position in the ranking have slightly greater complexity than the others (at least for the songs under consideration).

**Figure 5:**
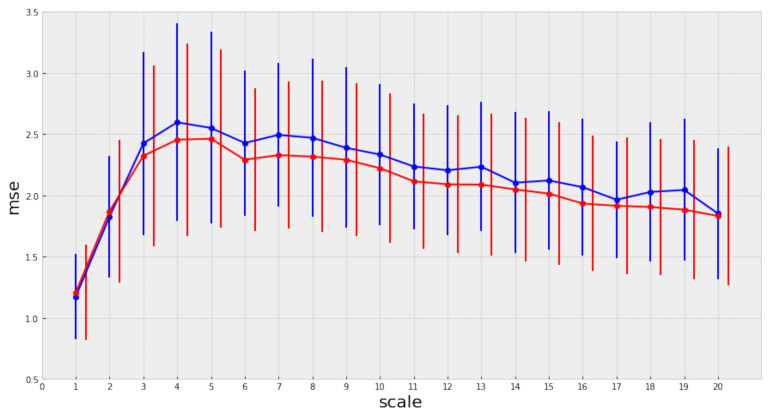
Complexity profile variance.

The mean complexity profile of the top songs is higher for each of the considered scales. However, there are many overlapping regions at the intra-group variance of the complexity profiles in the corresponding group distributions, as shown by Figure 5.

**Figure 6:**
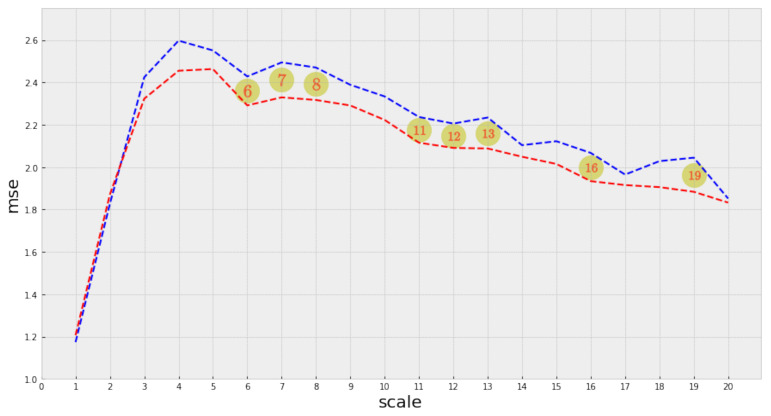
Statistically significant scales after the Welch test (level 0.05).

Due to the overlapping regions in the complexity profile distributions, it is necessary to evaluate the statistical significance of the differences we have previously identified between the complexity profiles of the top and non-top groups of songs, respectively. We use Welch’s test to evaluate the difference between two independent populations [32] and check for normality using the Shapiro–Wilk test [33]. The Welch’s test is a variant of Student’s *t*-test with the property of being more robust when the hypothesis of equal variance does not hold and when the sample sizes between the two populations are different. In our case, one of our groups has only 100 observations, the top group, for each year. In addition to the standard statistical test, it is important to note that we are facing a multiple hypothesis testing scenario (as we are simultaneously testing 20 scales). Then it becomes necessary to make a correction to take this into account. We use the Bonferroni correction [34] to adjust the significance results obtained with Welch’s test. In Figure 6, we present the results derived from the Welch test (before the Bonferroni correction).

After the Welch test, eight out of the twenty scales in the complexity profile resulted significant at level 0.05. It is important to note that the significant scales are distributed along with the profile’s range. Nevertheless, after applying the Bonferroni correction, the significance level dropped to 0.0025 (adjusted for 20 scales), at which none of the scales resulted as significant. Although not all scales in the complexity profile were statistically significant, the ones that were indeed significant are distributed along with the profile’s range: Table 3 and Appendix A.2 present detailed results.

Although the Bonferroni correction rendered all scales non-statistically significant, this is somehow an expected result given that many factors are contributing to the success or popularity of a song. Many of these factors are not even related to the musical properties of the songs but to external factors such as advertising expenses and social trends. Nevertheless, the analysis shows that the complexity profile of the top songs tends to be above that of the non-top songs for almost all the years of the studied period—a surprising fact considering the simplicity of our approach and the musical elements we are considering.

**Figure 7:**
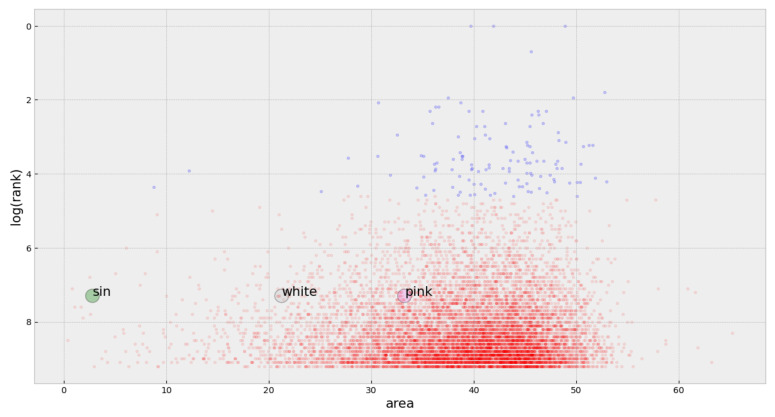
Area under the complexity profile for top songs (blue) and non-top songs (red) and its relation to log(rank) for the year 2000. For comparison purposes, white noise, pink noise, and the sinusoidal wave are included at an arbitrarily set rank.

In addition to the measured difference, the shape of the complexity profile provides an overview of some of the important characteristics of a system and its complexity scale relationship [18,35]. Nevertheless, to further investigate and compare the differences between the two groups of songs, we evaluate the relation between the total area under the complexity profile and the rank it obtained in the Billboard chart. We calculated the area under the complexity profile for all the songs in the two considered groups (top and non-top songs) to analyze this relation. We plotted these areas against the corresponding ranks (the logarithm of rank) for each song, Figure 7. As there is no rank information for the non-top songs, we assigned ranks for all these songs via a Monte Carlo simulation in which the overall shape of the distribution was invariant as the areas for each song kept fixed.

Figure 7 shows that the density of top songs tends to lay in the high side of the area spectrum, and the average area of a top song is always greater than the average area of a non-top song for all the considered years.

Interestingly, Figure 7 also suggests that area under the complexity profile of the most preferred songs tend to be in a specific range of the spectrum (not so low and not so high). Songs in the extremes of the spectrum are not widespread, thus indicating that there exists a preferred level of complexity (this same pattern was observed in all sampled years). Although we are not pursuing a predictive model for successful songs, Figure 7 lets us predict that if the calculated area for a given song is extremely low or extremely high, the corresponding song will certainly not be a well-ranked one.

This finding is somewhat related to [10], where the authors find a U-inverted relationship between complexity and preferences. Here, we found evidence that the area under the complexity profile of top songs is hardly located in the low or high extremes of the spectrum. However, as we do not have the exact rank positions for non-top songs, it becomes impossible to confirm the U-inverted shape. Nevertheless, our findings do not contradict the results described in [10].

We have also included in this figure the areas for the three signals (sinusoid, white noise, and pink noise) described in Section 3 as a reference to compare the difference between the complexity of a song and the complexity of the different signals.

## 5. Discussion

The meaning and quantification of complexity are under permanent discussion. Loosely speaking, one view suggests that the complexity of an object includes the effort needed to build an object’s description. Following this intuition, methods to estimate this description’s effort may include counting the number of object’s parts, assessing the relationship among these parts, or any applicable extensive counting procedure. To avoid the effects of prejudices in these counting processes, the notion of complexity, as intimately related to the information account in the object’s description, has been accepted [18,36,37]. Complexity is, therefore, a property of the object. Nevertheless, complexity brings the influence of the language used for the description and, more relevant for the scope of this work, the scale at which the object is observed. Thus, complexity shares objective and subjective aspects.

To consider the variations of complexity when the object is seen at different scales, the complexity profile [35] has been proposed. The complexity profile offers an overview of the object’s complexity interpreted at a range of scales.

Here, we have proposed a framework for analyzing the complexity associated with a song and relating this complexity to the listener’s preferences. Our findings suggest an association between complexity and preferences in the sense that preferred (well ranked) songs tend to have high complexity, at least for the considered songs and analyzed years. Furthermore, our results add some evidence suggesting the existence of an optimal level of complexity associated with our preferences.

In Figure 7, where we added the calculated areas for pink noise, white noise, and the sinusoid, it is worth noting that the area for the pink noise is close to that of the preferred songs, and this can be an explanation of why pink noise is sometimes used with relaxations purposes. Its complexity is higher than white noise but without the necessary elements to distract or catch our minds. We find this insight interesting as it opens the door for the study of relaxing sounds using techniques similar to the one we have described.

**Figure 8:**
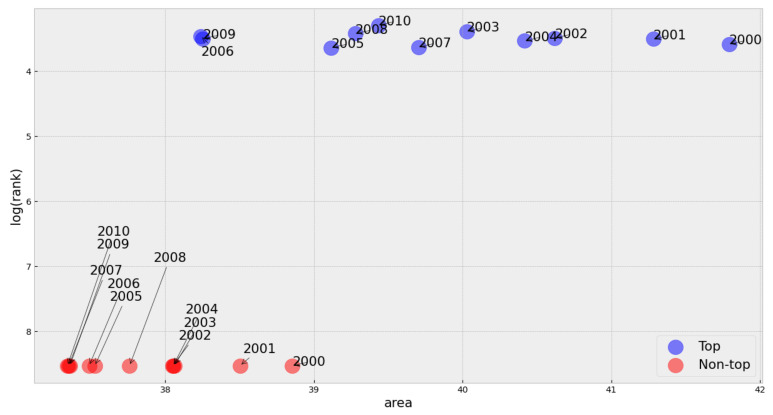
Average area vs. log(rank) for the two groups of songs in each year.

Furthermore, when computing the average area for each group, we observe that the mean area is higher for the top songs than the non-top areas. This comparison holds for every year in our sample and was evaluated using the Wilcoxon test for independent samples [38], as shown in Figure 8 (detailed analysis in Appendix A.6).

Although the framework presented here has some limitations and is far from describing a clear relationship (a predictive model) between complexity and preferences, it allows for a descriptive characterization of popular songs in terms of their multiscale complexity. Importantly, it provides a way to identify songs that will not be well ranked as they have extreme (low or high) complexity.

We used multiscale entropy to measure and characterize the complexity of a song’s melody when properly processed using standard music information retrieval (MIR) tools because this metric captures some of the critical aspects of a complex process in which we are particularly interested. Although MSE does not provide a complete description of the complexity of a process, nor is it the only alternative for measuring complexity, it does provide an interesting and innovative way to investigate the relationship between complexity and preferences when analyzing audio or music. We introduced this work intending to contribute to developing new methods to understand how the brain perceives and processes complex objects. Since audio represents many dimensions: time series, frequency, rhythm, number, and type of involved instruments, we decided to use audio signals (music) as our object of analysis. Due to this broad range of possibilities, there is no clear and unique definition of the informational content associated with a song nor a precise measure of its complexity. We hope that this work contributes to better frameworks and methodologies to analyze and understand complex processes such as music.

### 5.1. Limitations

We found a certain degree of association between multiscale complexity and popularity suggesting that the complexity of popular songs tends to be located in the high side of the range. Although the results presented in this article are not entirely conclusive in the sense of providing a clear relation between complexity and preferences, this can be associated with the following:

- The associated factors involved for a song to become popular are more than we can afford to consider in a study such as this.
- Many of the involved factors are not directly associated with the complexity of the song, for example, social trends, cultural biases, spending on advertising, and sample design biases, etc.

These exogenous factors make it difficult to compute an unbiased estimation of the relationship between music complexity and its corresponding public preferences. We believe, however, that there exists a level of music complexity where most people will find this music as pleasant. This "optimal" level of music complexity can be estimated with the methods presented.

### 5.2. Future Work

The study can be extended to make a complexity metric that accounts for more musical features. Here, we limited the analysis to pitch sequences to construct a time series and only to the most relevant pitch element. As the database includes the complete chromatogram for each song, it is possible to select different combinations of pitch elements according to their relevance. This generalization could consist of:

1. Consider a complexity profile for each level of relevance.
2. Construct a weighted average considering the distinct pitch classes involved in each time segment and calculate the complexity profile of this weighted series.

In addition to the pitch elements, the database includes the timbre and loudness elements. An identical treatment to the one described for pitch might be helpful to generate the corresponding complexity profiles. Different combinations of musical elements will allow for a richer approximation of music. One practical and interesting application of the framework we have presented is to use the complexity profiles to improve music recommender systems in streaming platforms. It is even possible to use the complexity profile to generate new music by following specific complexity patterns associated with customers’ preferences.

An analysis of the complexity profiles between genres would be illuminating. It would be interesting to find out if there is a relevant difference between two songs that belong to the same genre, but one is popular (top), and the other is not (non-top). Furthermore, to investigate if each musical genre has a characteristic complexity profile. In addition to these experiments, the complexity profile could be used as a feature in predictive models, for example, trying to predict the genre of a song given its complexity profile. More elaborate processing and treatment are necessary to carry out this analysis.

In future work, it would also be interesting to compare different complexity metrics to determine the degree of similarity between MSE and other metrics for the same analysis. Furthermore, it would be important to evaluate how robust our results are with respect to parameter changes in the pre-processing steps, the musical elements considered or in the sampling design.

Finally, it is important to remark that music also has therapeutic properties. Our analysis found that pink noise has a complexity close to the preferred songs, making this a possible guide for creating music with properties in between the spectrum of pink noise and popular music that could have better results in musical therapies. Some rehabilitation therapies use musical stimuli to treat memory and speech-related problems [39,40,41]. A complexity analysis relating sensory stimuli and the corresponding patient’s response can help identify and select the stimulus for the appropriate treatment.

## Figures and Tables

**Table 1:** SE for a sinusoid signal and pink and white noises.

Signal	Sample Entropy (SE)
Sinusoid	0.4675
Pink noise	1.7735
White noise	2.1752

**Table 2:** MSD database technical components.

Component	Description
Key	Estimation of the key the song is in
Loudness	General loudness of the track
Segment_pitches	Chroma features for each segment
Segments_timbre	MFCC-like features for each segment
Segments_loudness_max	Max loudness during each segment

**Table 3:** Difference and statistical significance (year 2000).

Scale	Difference	*p*-Value	Welch ( α=0.05 )	Bonferroni ( α=0.0025 )
6	0.1369	0.014	Yes	No
7	0.1651	0.004	Yes	No
8	0.1532	0.024	Yes	No
11	0.1213	0.017	Yes	No
12	0.1148	0.044	Yes	No
13	0.1460	0.011	Yes	No
16	0.1333	0.022	Yes	No
19	0.1611	0.017	Yes	No

**Figure A1:**
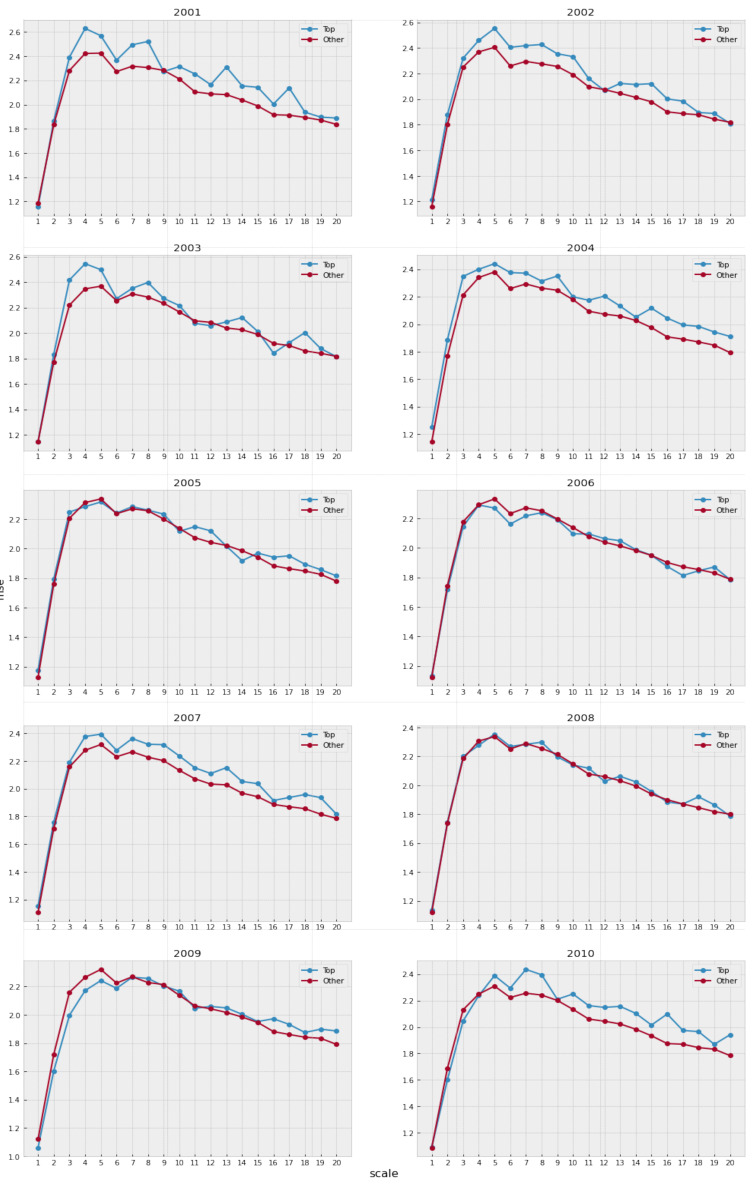
Complexity profiles (2001-2010).

**Figure A2:**
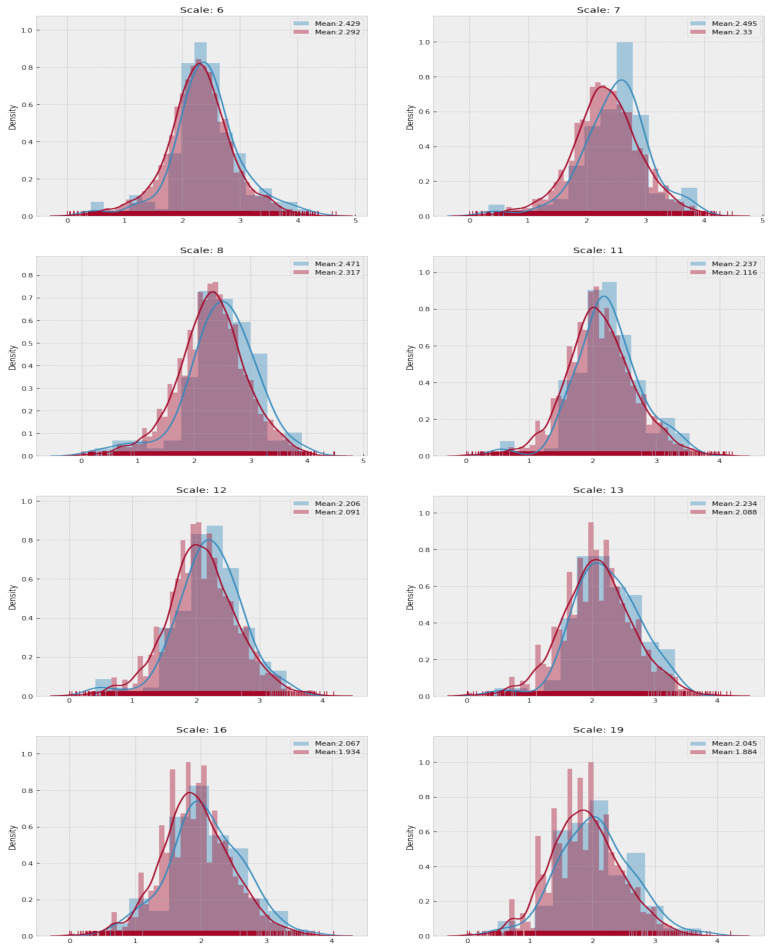
Statistically significant distributions (year 2000).

**Figure A3:**
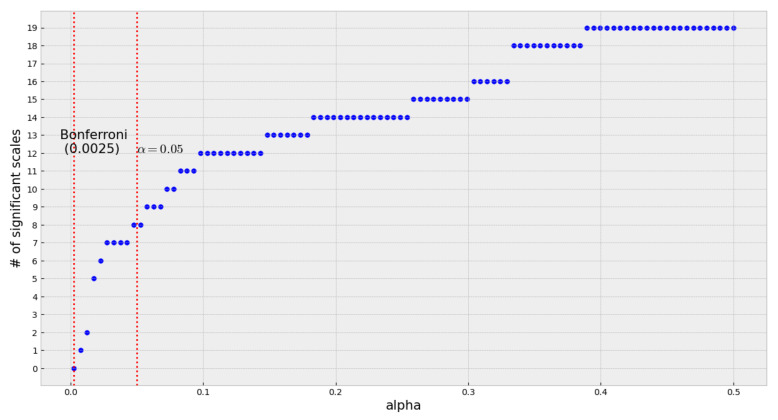
Statistical level needed to achieve significant scales.

**Figure A4:**
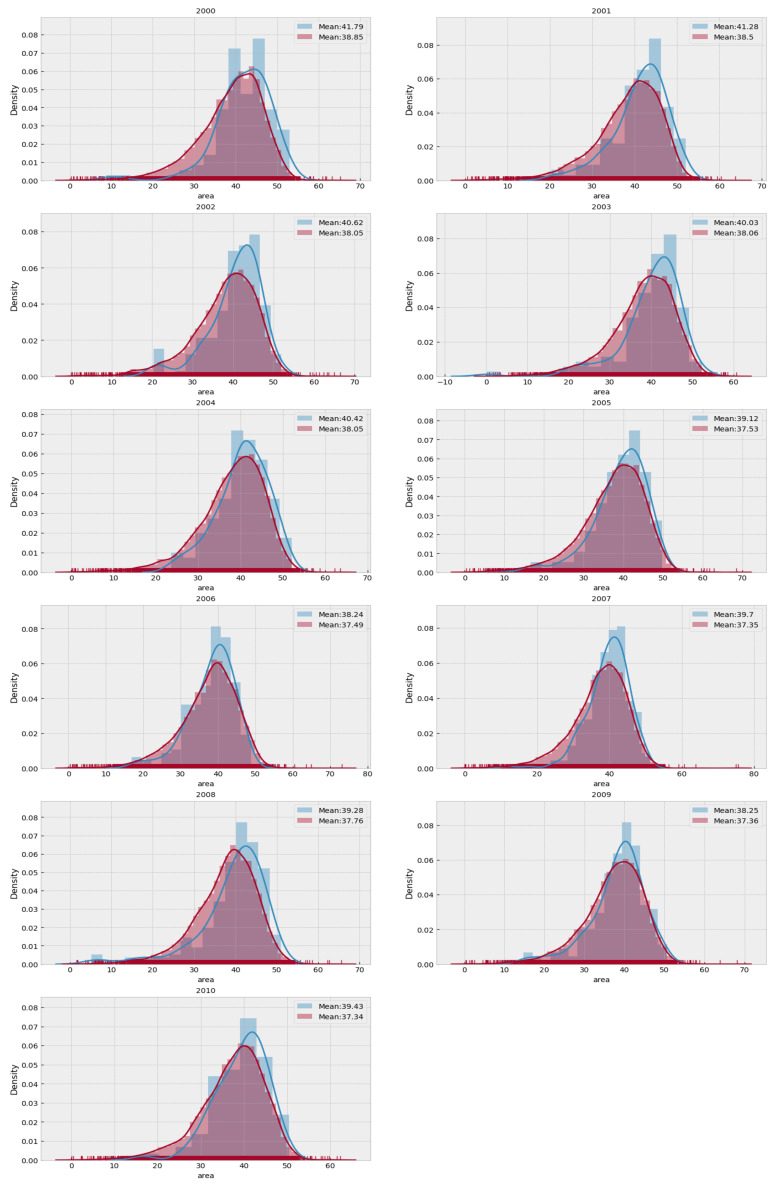
Average area vs. log(rank) for the two groups of songs in each year.

## Data Availability

Data from MSD can be found at https://aws.amazon.com/datasets/million-song-dataset/ (accessed on 30 September 2021). The year-end HOT 100 Billboards are available at https://www.billboard.com/charts/year-end/2020/hot-100-songs (accessed on 30 September 2021).

## Abbreviations

The following abbreviations are used in this manuscript:

MSE	multiscale entropy
SE	sample entropy
fMRI	functional magnetic resonance imaging
MSD	million song dataset
MIR	music information retrieval

## Appendix A

### Appendix A.3. Statistically Significant Differences in Scale (2001–2010)

There were no statistically significant scales for the years 2005, 2006 and 2008. This can be by assessed observing that the respective profiles are almost completely overlapping. For the rest of the years, the statistical significance is presented in the following tables:

**Table A1:** Statistical significance 2001.

Scale	Calculated Difference	*p*-Value
4	0.2066	0.0102
5	0.1437	0.0341
7	0.1771	0.0014
8	0.2158	0.0015
11	0.1481	0.0049
13	0.2281	0.0002
15	0.1544	0.0217
17	0.2249	0.0006

**Table A2:** Statistical significance 2002.

Scale	Calculated Difference	*p*-Value
5	0.1480	0.0269
6	0.1458	0.0061
7	0.1236	0.0307
8	0.1513	0.0182
10	0.1415	0.0305
15	0.1418	0.0428

**Table A3:** Statistical significance 2003.

Scale	Calculated Difference	*p*-Value
3	0.1979	0.0032
4	0.1978	0.0074
18	0.1434	0.0447

**Table A4:** Statistical significance 2004.

Scale	Calculated Difference	*p*-Value
1	0.1071	0.0009
2	0.1192	0.0091
3	0.1353	0.0282
6	0.1163	0.0177
12	0.1319	0.0391
15	0.1416	0.0132
16	0.1366	0.0066
17	0.1037	0.0367
18	0.1131	0.0386
20	0.1176	0.0260

**Table A5:** Statistical significance 2007.

Scala	Calculated Difference	*p*-Value
1	0.0465	0.0414
7	0.0955	0.0449
8	0.0936	0.0437
9	0.1154	0.0174
10	0.1039	0.0354
13	0.1243	0.0043
18	0.1019	0.0328
19	0.1197	0.0107

**Table A6:** Statistical significance 2009.

Scale	Calculated Difference	*p*-Value
1	−0.0598	0.0050
2	−0.1173	0.0002
3	−0.1628	0.0002
16	0.0910	0.0465

**Table A7:** Statistical significance 2010.

Scale	Calculated Difference	*p*-Value
7	0.1798	0.0145
8	0.1531	0.0173
16	0.2233	0.0094

### Appendix A.4. Shapiro–Wilk Test for Normality in Scale Distributions (2000–2010)

The Shapiro–Wilk test was used to evaluate the normality assumption in the scale distributions used in Welch’s test. When the sample was too large (as in the case of all non-top groups of songs, ∼10,000 samples), the test rendered non-significant results, but for large samples, the normality assumptions are not strongly required as they are for small samples. The *p*-values presented in the following tables correspond to the small samples (top-songs).

**Table A8:** Shapiro–Wilk test. *p*-values per scale and year (2000-2005).

Scale	2000	2001	2002	2003	2004	2005
1	0.9329	0.0936	0.0326	0.1685	0.2854	0.0633
2	0.0365	0.1406	0.2010	0.1104	0.1980	0.0006
3	0.8561	0.6597	0.8340	0.0013	0.5701	0.7112
4	0.0699	0.1221	0.0936	0.0879	0.6430	0.4746
5	0.5008	0.3178	0.5555	0.3835	0.1539	0.2213
6	0.0006	0.0065	0.0163	0.0011	0.2359	0.0341
7	0.0020	0.0856	0.4961	0.0031	0.1052	0.2203
8	0.0021	0.4071	0.2640	0.0097	0.5182	0.6734
9	0.1027	0.1666	0.8300	0.0160	0.1497	0.3567
10	0.0455	0.9184	0.4900	0.7766	0.0203	0.8399
11	0.0153	0.0454	0.4747	0.1271	0.9733	0.6351
12	0.0131	0.1530	0.8234	0.0076	0.0030	0.9613
13	0.1261	0.6955	0.2467	0.6120	0.7281	0.1045
14	0.8757	0.1543	0.6581	0.1633	0.0269	0.5477
15	0.3005	0.1063	0.9445	0.6983	0.2720	0.5705
16	0.8187	0.1645	0.0264	0.3736	0.0928	0.7060
17	0.0322	0.0705	0.4356	0.2320	0.0775	0.6569
18	0.3397	0.8494	0.9125	0.3709	0.1332	0.6508
19	0.7292	0.7102	0.0528	0.6223	0.4121	0.6581
20	0.6156	0.1075	0.3672	0.4147	0.0047	0.4603

**Table A9:** Shapiro–Wilk test. *p*-values per scale and year (2000-2005).

Scale	2006	2007	2008	2009	2010
1	0.4237	0.0457	0.5723	0.4247	0.6414
2	0.1894	0.7549	0.1976	0.5587	0.2651
3	0.1428	0.1868	0.3652	0.2921	0.0346
4	0.4861	0.5645	0.0102	0.5316	0.6629
5	0.1318	0.7352	0.0313	0.1480	0.0420
6	0.1656	0.0006	0.0016	0.0006	0.0044
7	0.1119	0.0037	0.0021	0.0040	0.3237
8	0.5568	0.0335	0.0025	0.1747	0.1008
9	0.0157	0.3449	0.0187	0.2692	0.0422
10	0.1804	0.6223	0.0324	0.4781	0.3087
11	0.3535	0.0303	0.0002	0.6112	0.0283
12	0.8280	0.3112	0.0132	0.2647	0.9715
13	0.8021	0.5907	0.0091	0.3962	0.3695
14	0.1404	0.0152	0.0005	0.1899	0.7427
15	0.7298	0.5955	0.0001	0.6707	0.1390
16	0.5348	0.2038	0.0025	0.4316	0.1962
17	0.0560	0.1144	0.0012	0.0534	0.0428
18	0.0923	0.5496	0.1424	0.2645	0.1992
19	0.2156	0.1237	0.0576	0.0001	0.7480
20	0.7259	0.5339	0.0857	0.0367	0.2382

### Appendix A.6. Statistically Significant Differences for Area under the Complexity Profile (2000–2010)

**Table A10:** Significance test for area distribution between top and non-top songs in each year (Wilcoxon test, α=0.05 ).

Year	Difference	*p*-Value	Significant
2000	2.939693	0.000003	Yes
2001	2.780737	0.000030	Yes
2002	2.569101	0.000033	Yes
2003	1.967215	0.000193	Yes
2004	2.362849	0.000177	Yes
2005	1.589775	0.000940	Yes
2006	0.751036	0.143811	No
2007	2.353117	0.000004	Yes
2008	1.518561	0.000003	Yes
2009	0.892549	0.032301	Yes
2010	2.091714	0.006654	Yes
